# Abnormal Presentation of Bartonella henselae Encephalopathy in a Pediatric Patient

**DOI:** 10.7759/cureus.43535

**Published:** 2023-08-15

**Authors:** Emily J Etter, Spencer J Trivitt, Benjamin L Bosse, Alison McWilliams

**Affiliations:** 1 Pediatrics, University of Nevada, Reno School of Medicine, Reno, USA; 2 Radiology, University of Nevada, Reno School of Medicine, Reno, USA

**Keywords:** gait ataxia, neurology, infectious disease, titers, seizures, encephalopathy, pediatric, cat scratch disease, bartonella henselae

## Abstract

Cat scratch disease (CSD) is a zoonotic infection caused by the transmission of gram-negative bacteria *Bartonella henselae* through a scratch or bite of a feline carrying *B. henselae-*infected fleas. CSD often presents clinically as a self-limited flu-like infection with painful regional lymphadenopathy appearing one to two weeks following initial transmission. However, a growing body of literature highlights abnormal presentations of *Bartonella* infections within the pediatric population. In this case report, we describe an atypical presentation of a *B. henselae* infection in an 11-year-old female with seizures, prolonged encephalopathy, agitation, and truncal instability. With an atypical presentation, a delay in diagnosis can result in potentially permanent organ damage, particularly as traditional empiric antibiotics fail to cover *Bartonella* infections. As such, proper treatment and complete resolution of symptoms require astute clinical recognition to make the correct diagnosis promptly.

## Introduction

Cat scratch disease (CSD) has an annual incidence of 22,000 across the United States, most frequently affecting pediatric patients in the fall and winter months [1,2]. It most often presents as a mild, self-limited, painful regional lymphadenopathy associated with a flu-like illness and fevers one to two weeks following the transmission of *Bartonella henselae* through a skin lesion from a feline [3]. As infections with *B. henselae* are generally self-limited, the treatment is largely supportive [1-3]. However, there are more severe clinical presentations outlined in the literature that require prolonged and more extensive treatment. *B. henselae* is a zoonotic gram-negative bacterium horizontally transferred between felines by the cat flea, *Ctenocephalides felis* [3,4]. These fleas are harbored asymptomatically in over 50% of cats [2].

## Case presentation

Here, we present a case of an 11-year-old female with a past medical history of oppositional defiant disorder and sensory integration disorder who presented to the emergency department (ED) after falling backward out of a bunk bed and hitting her head. According to her parents, this was followed by a 30-second episode of full body shaking suspected to be a tonic-clonic seizure with no evidence of tongue biting or urinary incontinence. Her parents reported no other unusual activity or uncharacteristic behavior surrounding the event or in the days prior. The patient, her parents, older brother, and family cat had been on a cross-country RV road trip for the past few months. 

On physical exam in the ED, she was tachycardic in the 150s and a presumed postictal state with agitation and confusion. There were no lacerations or edema present. A complete blood count (CBC) with differential, complete metabolic panel, magnesium, urinalysis, and urine toxicology screen were all within normal limits other than a slightly elevated glucose at 122 and benzodiazepines in her urine, which had been administered in the ED for her suspected seizure. Electrocardiogram (EKG) demonstrated sinus tachycardia without ST changes, and a head and neck CT without contrast indicated no intracranial or cervical pathology. The patient's mental status continued to be altered from her baseline with agitation and tachycardia worrisome for a prolonged postictal state. She was admitted to the pediatric intensive care unit (PICU) where a brain MRI, an infectious work-up with a cerebral spinal fluid (CSF) encephalitis/meningitis panel, and blood and urine cultures were completed. The results of the initial laboratory tests were all within normal limits. She also had an electroencephalography (EEG) demonstrating slow to moderate amplitude delta waves across bilateral hemispheres but more prominent anteriorly with no interictal epileptiform discharges or sharp wave complexes seen, most consistent with encephalopathy. 

Without intervention, the patient began to return to baseline over the next few days. She was discharged once she was close to her baseline mental status but was noted to have some mild gait instability and was provided a clear return to activity instructions and return to ED precautions. On the day following discharge, the patient returned to the hospital for a seizure-like activity, subjective fevers, and hypersomnolence. She had another seizure episode on hospital day two described as total body rigidity with tremors, a glazed state with deviated eyes, tachycardia in the 120s, and hypoxia down to 67%, which responded to nasal cannula oxygen. She did not have any tongue lacerations or incontinence. These seizures were unable to be recorded through EEG, which only showed continued encephalopathic slow delta wave changes. A pediatric neurologist was consulted who began the patient on levetiracetam for her seizure activity in the interim until the primary cause was determined. She continued to experience waxing and waning behavioral changes mainly presenting as episodes of agitation, post-concussive symptoms of nausea, vomiting, headaches, and truncal instability, described as unstable posturing and poor balance, especially with walking. A repeat MRI was again noted to be normal. Psychiatry was consulted for her intermittent agitation and determined that no primary psychiatric disorders were contributing to her ongoing symptoms. A pediatric infectious disease specialist was consulted who recommended a wider array of specialized tests. All were negative except a markedly elevated *B. henselae* immunoglobulin G (IgG) titer at >1:1024 indicating a recent or active infection with *B. henselae*. Her immunoglobulin M (IgM) titers were <1:16. Per infectious disease recommendation, baseline liver function tests and CBC were obtained, and she was prescribed a 14-day course of doxycycline and rifampin. The patient was also provided a month's supply of levetiracetam and, as needed, diazepam rectal gel for breakthrough seizures. Baseline CBC showed eosinophilia at 18% with a reference range of 0-4%, and absolute eosinophils were elevated at 1.5 K/uL, with a reference range of 0-0.47 K/uL. Liver function tests were within normal limits. Blood and CSF cultures were negative for *B. henselae*. Ophthalmology was consulted and determined that she was negative for acute optic neuritis. 

The patient was seen by pediatric infectious disease three months after discharge from the hospital and was back to her baseline. She finished the 14-day course of rifampin but was only able to finish four days (seven of 26 pills) of the prescribed doxycycline due to vomiting. The patient did not complete repeat laboratory testing following the completion of the antibiotic course. There was no documentation about levetiracetam doses or diazepam rectal gel in the follow-up note.

## Discussion

CSD commonly presents as a self-limited flu-like illness with regional lymphadenopathy following the transmission of *B. henselae* through the skin [1-4]. While CSD is the most common clinical presentation of a *B. henselae* infection, other well-documented manifestations include bacillary angiomatosis, Parinaud oculoglandular syndrome, and neuroretinitis [4]. More rarely, immunocompetent patients can present with encephalitis, meningoencephalitis, endocarditis, hepatic, splenic, and paravertebral abscesses, and osteomyelitis [4]. Encephalopathy secondary to CSD occurs one to six weeks following the onset of lymphadenitis and can initially present with status epilepticus [5,6]. The exact pathophysiology of how* B. henselae* causes encephalopathy is not completely understood. In immunocompetent patients, occasionally, pleomorphic rod-shaped bacilli can be seen within the vascular endothelium using a special Warthin-Starry stain, suggestive of vasculitis. In other patients, it is hypothesized that there is a direct bacterial invasion into the central nervous system, which causes granulomatous inflammation affecting the meninges and secondarily causing cerebral edema [7-9]. Most patients with encephalopathy make a full recovery with no lasting deficits, but there are reported cases of ongoing seizures, focal deficits, dementia, and, rarely, death [5,6,10]. A two-and-a-half-year longitudinal study conducted in California concluded that *B. henselae* was the cause of encephalopathy in less than 1% of pediatric and adult patients [11]. A retrospective study analyzing seven years of pediatric data in Houston, Texas, suggests that approximately 3% of pediatric encephalopathy cases are due to *B. henselae* and that it is one of the least frequent infectious causes tested for during an encephalopathy work-up [12].

*Bartonella* species are difficult to culture. Thus, the diagnosis of *B. henselae* is most often made through serology using indirect fluorescent assay (IFA) or enzyme-linked immunosorbent assay (ELISA) [3,4]. Serology is a sensitive test but lacks specificity due to widespread previous asymptomatic exposure within the general population [3,4,13]. Thus, to increase sensitivity, current guidelines state that an IgG titer >1:256 or a four-fold increase in titers strongly suggests an active or recent infection. A positive IgM titer is also supportive of a current *Bartonella* infection but is more rarely found as IgM elevations are brief. An IgG titer between 1:64 and 1:256 represents a possible infection, and repeat titers in 10-14 days looking for an increase or other supporting testing, such as serum, and/or CSF polymerase chain reaction(PCR) testing should be performed. An IgG titer less than 1:64 does not support a current *Bartonella* infection [3,4,12,14].

Treatment for mild or uncomplicated CSD is frequently supportive as it is a self-limiting disease [1-3]. Rarely, oral azithromycin has been used in select cases based on clinical judgment and the patient’s individual presentation to provide a more rapid resolution of lymphadenopathy [3]. Antibiotic use is more widely accepted for more severe complications or neurologic manifestations of *Bartonella* infections. Most frequently, a prolonged course of oral erythromycin or doxycycline plus rifampin has been effective in most patients [4]. Patients with a neurological complication caused by *Bartonella* require antibiotics for three to four months, excluding encephalitis that is often treated with a two-week course [13].

## Conclusions

This patient demonstrated multiple characteristics that did not fit a classic clinical picture for CSD. She initially presented with a witnessed tonic-clonic seizure, which then progressed to prolonged tachycardia, agitation, truncal instability, and EEG findings consistent with encephalopathy. Through diagnostic testing, the causative agent was found to be *B. henselae*. This atypical presentation highlights the importance of maintaining a broad list of differential diagnoses. *Bartonella* infections are treated with multiple antibiotics, including rifampin, which is not generally included in traditional empiric antibiotics. As such, a prompt diagnosis of an atypical presentation is required in order to treat patients with antibiotic regimens that cover *Bartonella* to prevent potentially permanent organ damages. For atypical non-focal neurological presentations in pediatric patients, serum and CSF titers for *Bartonella* should be considered.
